# Epigenetic differences between wild and cultivated grapevines highlight the contribution of DNA methylation during crop domestication

**DOI:** 10.1186/s12870-024-05197-z

**Published:** 2024-06-06

**Authors:** Alberto Rodriguez-Izquierdo, David Carrasco, Lakshay Anand, Roberta Magnani, Pablo Catarecha, Rosa Arroyo-Garcia, Carlos M. Rodriguez Lopez

**Affiliations:** 1 Centro de Biotecnología y Genómica de Plantas (CBGP-INIA), CSIC - Universidad Politécnica de Madrid, Campus Montegancedo, Madrid, Spain; 2 Environmental Epigenetics and Genetics Group (EEGG), Department of Horticulture, College of Agriculture, Food and environment, University of Kentucky, Lexington, KY USA

**Keywords:** Domestication, Epigenomics, Grapevine, EpiGBS, Wild, Cultivated, Methylation, Epigenetic memory

## Abstract

The domestication process in grapevines has facilitated the fixation of desired traits. Nowadays, vegetative propagation through cuttings enables easier preservation of these genotypes compared to sexual reproduction. Nonetheless, even with vegetative propagation, various phenotypes are often present within the same vineyard due to the accumulation of somatic mutations. These mutations are not the sole factors influencing phenotype. Alongside somatic variations, epigenetic variation has been proposed as a pivotal player in regulating phenotypic variability acquired during domestication. The emergence of these epialleles might have significantly influenced grapevine domestication over time. This study aims to investigate the impact of domestication on methylation patterns in cultivated grapevines. Reduced-representation bisulfite sequencing was conducted on 18 cultivated and wild accessions. Results revealed that cultivated grapevines exhibited higher methylation levels than their wild counterparts. Differential Methylation Analysis between wild and cultivated grapevines identified a total of 9955 differentially methylated cytosines, of which 78% were hypermethylated in cultivated grapevines. Functional analysis shows that core methylated genes (consistently methylated in both wild and cultivated accessions) are associated with stress response and terpenoid/isoprenoid metabolic processes. Meanwhile, genes with differential methylation are linked to protein targeting to the peroxisome, ethylene regulation, histone modifications, and defense response. Collectively, our results highlight the significant roles that epialleles may have played throughout the domestication history of grapevines.

**Supplementary Information:**

The online version contains supplementary material available at 10.1186/s12870-024-05197-z.

## Background

Domestication syndrome is a phenomenon observed in crops. This results in a suite of traits that distinguish cultivated genotypes from their wild progenitors, including changes in morphology, physiology, and phenology that make them more amenable to cultivation. Thanks to the abundance of archaeobotanical, ecological and genetic information available for a handful of economically important seed propagated crops, the domestication syndrome has been well-documented in these species [1, 2]. However, less is known about the domestication trajectories of vegetatively propagated crops [2]. One of the main advantages of vegetative propagation is that it allows for the preservation of desirable traits from one generation to the next. This is because when a plant is propagated vegetatively, the offspring is genetically identical to the parent plant [2–4]. This means that desirable traits such as disease resistance, yield, and flavor can be maintained over many generations. This contrasts with sexual reproduction, where traits can be lost or diluted through the process of genetic recombination. The type of propagation used during domestication can result in diametrically opposed domestication syndromes. For example, while the use of vegetative propagation has been shown to negatively affect the capacity for sexual reproduction via the accumulation of mutations in genes associated to flower development, self-fertilization, and seed development, which lead to the production of self-fertilized fruits, flowering asynchrony, and lower seed viability [2, 5, 6]; crops domesticated by sexual reproduction, tend to present larger seeds, synchronic flowering and pollinator dependent fertilization [2].

*Vitis vinifera* is a perennial woody liana belonging to the Vitaceae family. The species is divided into two different forms principally based on their reproductive system and whether it is a cultivated or a wild form. Wild grapevines (*V. vinifera* ssp. *sylvestris*), are commonly dioecious plants [7], and are naturally distributed across Asia and Europe. Cultivated grapevines (*V. vinifera* ssp. *vinifera*) mainly produce hermaphrodites flowers, and are broadly cultivated across the world, both for grape production to be consumed as a fruit, and for winemaking, grape juice or other derived products [8].

Although viticulture started at the Paleolithic age as a food source in Europe from wild accessions [9], there is evidence that the use of grapes by humans to produce wine started near to the seventh millennium BC [10]. This significantly influenced the domestication of grapevines by selecting varieties that produce a particular fruit quality and larger berries [7, 8]. It is believed that such selection occurred using vegetative propagation by cuttings to enhance the preservation of phenotypes of interest [7, 8, 11], which in turn had a negative effect on the crop tolerance to biotic and abiotic stresses. For example, populations of wild grapevines in North Africa and coastal regions of Northern Spain shown better adaptation to salt stress than cultivated grapevines [12, 13], while wild accessions from Germany, Iran and Georgia show higher resistance to mildew infections [14–17]. Moreover, despite the use of vegetative reproduction to maintain a desired genotype, the use of asexual reproduction in grapevine has resulted in novel phenotypes appearing within the same variety [5] and same vineyard [18]. Such phenotypic variants are frequently found in vegetatively propagated crops and often make up a significant portion of the cultivated varieties. Although a genetic basis is often presumed to be the reason for the noticeable differences in traits observed, epigenetic modifications have also been proposed to play an important role [19–22].

Epigenetic modifications are potentially heritable changes in gene expression and function that give rise to a certain phenotype without changes to their underlying DNA sequence [23]. The most studied type of epigenetic modification is DNA methylation, defined here as the addition of a methyl group to the carbon 5 of cytosines [24]. DNA methylation can be transient and can change rapidly during the life span of a cell or organism, or it can be essentially permanent once set early in the development of the embryo. Moreover, recent research has shown that DNA methylation epialleles can be used as an epimutation clock to enable the phylogenetic reconstruction of the recent history of vegetatively propagated plants [25], highlighting their heritability and potential contribution to plant diversification.

Several studies suggest that DNA methylation might have played a role in plant domestication. This was first made evident in studies analyzing the effect of polyploidy on DNA methylation in hybrid plant species [26], including crops such as wheat [27] and cotton [28]. For instance, in hexaploid wheat, the removal of the D sub-genome leads to a genome-wide reduction in DNA methylation. A reduction that is reversed in the resynthesized hexaploid wheat [27]. More recently, detailed analysis of DNA methylation in rice [29] and tomato [30] has shown that domesticated cultivars present lower levels of DNA methylation than their wild counterparts. Moreover, multiple studies have shown that differentially methylated regions associated to domestication overlap with genes linked to traits known to be under selection during domestication of soybean [31], tomato [30], maize [32], and cotton [28]. However, our understanding of how these epigenetic modifications were utilized or inadvertently altered during the domestication process remains rudimentary. Specifically, the effects of domestication on DNA methylation have been infrequently studied in perennial crops [33].

In this study, we employ reduced representation bisulfite sequencing to characterize and compare the methylomes of wild and cultivated grapevine accessions grown under common garden conditions. We aim to determine whether the domestication process has influenced methylome modeling in grapevine. We hypothesize that the combination of phenotype selection and vegetative propagation during grapevine domestication has led to distinctive methylome characteristics in cultivated grapevines, which significantly differ from those in wild accessions, such as higher levels of DNA methylation. Furthermore, we speculate that the epialleles observed in cultivated accessions could be linked to phenotypic traits traditionally associated with domesticated crops.

## Methods

### Experimental design

Single ortets from 10 *V. vinifera* ssp. *vinifera* cultivars (Albillo Mayor, Allaren, Bocalilla, Brujidera, Espadeiro, Graciano, Heben, Jaen, Marfal and Zalema) and 8 *V. vinifera* ssp. *sylvestris* accessions (CA2.9b, CA4.1, CA5.1, H7.8, O1.5, S1.7, SE3.4 and VI3.4) kept in a in vivo grapevine germplasm bank located at IMIDRA (Instituto Madrileño de Investigación y Desarrollo Rural, Agrario y Alimentario, Alcalá de Henares, Madrid, Spain), were used to generated triplicate ramets from dormant wood cuttings. All ortets were generated from material originally collected from different locations in continental Spain (see Supplementary Table S1 for more information). Accession unique identifiers (Supplementary Table S1) are denoted by an alphanumeric code e.g. ESP080-BGVCAMXXXX, where XXXX indicates a number unique to each accession. All plants were originally identified by Dr. Alejandro Benito Barba. Cuttings were collected in winter, January 2021, at dormancy stage, from ortets planted on the same parcel. Cuttings were disinfected with tebuconazole and treated with rooting hormone (indole-butiric acid (IBA) 5 g/L), and then potted in individual containers (1.6 L truncated conic pots with drain sink) filled with potting mix 70% peat / 20% perlite / 10% sand. All propagules were then placed under the same conditions (light 16 h 21º C - dark 8 h 16º C) in a single growth chamber, with all the cuttings distributed randomly along the growth chamber. After budbreak, the second and third fully open leaves were collected and immediately snap-frozen using liquid nitrogen and preserve at -80º C until DNA extractions.

### DNA extraction and epiGBS protocol

Total DNA was extracted from all samples using the QIAGEN DNEasy Plant Mini Kit (Qiagen N. V., Hilden, Germany) following manufacturer’s instructions. DNA samples concentrations were determined using a Fragment Analyzer High Sensitivity DNA kit (Agilent). Sample concentration was standardized to 10 ng/ul.

Reduced representation bisulfite sequencing (RRBS) libraries were prepared for all samples following the epiGBS2 protocol [34, 35] by digesting 100 ng of DNA with restriction enzymes *Nsi*I and *Csp*6I (New England Biolabs, UK). Individually barcoded hemimethylated adapters, designed for the resulted restriction sites, were ligated to the resulting restriction products and amplified using PCR. Individual libraries generated from each sample were equimolarly mixed into two libraries which were sequenced using two Illumina HiSeq 2500 150 bp paired-end runs by NovoGene USA.

### Bioinformatic analysis

All bioinformatics tools included below used their default parameters unless specific parameters are presented. Sequencing library quality was checked using FastQC v0.11.8. A custom workflow was built to adapt the epiGBS workflow [34–36] to our data. Firstly, demultiplexing was performed in order to ensure the structure of the adapters to identify the samples [35], and a fastq-filter was performed using Stacks v2.55 [37]. The demultiplexed sequences from the triplicates from each accession were pooled to form a unique sample. Paired-end sequences were merged using PEAR v0.9.6 [38]. Alignment and methylation calling were performed with Bismark v0.23.0 [39] using the reference genome of *Vitis vinifera* L. PN40024 v4.1 [40]. Sequencing depth, coverage, and methylation differences between wild and cultivated accessions were visualized using ChromoMap R v1.0.0 [41].

Global differences in DNA methylation were visualized using hierarchical clustering and principal component analysis (PCA) performed using MethylKit R Package v1.16.1 [42] on the calculated percentage of methylation in all methylated cytosines present in at least four of the accessions. The percentage of total methylation was compared between cultivated and wild accessions in each context (CG, CHG and CHH (where H = A, T,C)) using T-test, after testing for normality in the data using Kolmogorov-Smirnoff Test, considering significant differences when *p*-value < 0.01. Finally, differentially methylated cytosines (DMCs) were identified using the methylKit R package v1.16.1 [42]. Cytosines were considered differentially methylated between wild and cultivated accessions when the observed difference in methylation was more than 25% and *p*-value < 0.01. To reduce the effect of genetic mutations on differential methylation data, for a genomic location to be included in the differential methylation analysis, such location must have a cytosine in a minimum of four samples per group and the location must have been sequenced to a minimum coverage of 10X. Additionally, a second more stringent filtering was implemented by identifying all genomic locations containing a SNP using the epiDiverse - SNP pipeline (available at “https://github.com/EpiDiverse/SNP”). Then, all epialleles located in genomic locations containing a SNP were removed from the analysis and hierarchical clustering was performed using all remaining epialleles.

To determine if DNA methylation patterns associated to the geographic origin of wild accessions were present, we performed a comparative analysis following the premises of De Andrés et al., (2012) [43]. For this, the methylation information gathered from wild accessions was filtered for epialleles associated to single nucleotide polymorphism as described above. Both the remaining epialleles and the SNPs identified using epiDiverse - SNP pipeline were used for hierarchical clustering analysis.

Protein coding genes presenting at least one methylated cytosine within 1000 bp of the transcription start site were deemed methylated. The annotated genome PN40024 v4.1 was used to determine the genic location (promoter, intro, exon) of methylated cytosines identified within genes. Then methylated genes were divided into 6 groups based on the type of methylation observed: 1. Core methylated genes, i.e., genes presenting unchanged methylated cytosines both in wild and cultivated accessions (CMCs); 2. Genes presenting CMCs and hypermethylated differentially methylated cytosines (DMCs) in cultivated compared to wild accessions; 3. genes presenting CMCs and hypomethylated DMCs in cultivated compared to wild accessions; 4. genes presenting CMCs and both hypomethylated and hypermethylated DMCs; 5. genes presenting hypermethylated DMCs in cultivated compared to wild accessions; and 6. genes presenting hypomethylated DMCs in cultivated compared to wild accessions. As above, DMCs associate with a SNP were removed from the analysis using the epiDiverse - SNP pipeline. Gene Ontology (GO) analysis was implemented with GOstats [44] and rrvgo [45] package in R, for each of these groups using all genes sequenced (i.e., presenting at least one read overlapping with a window of 1000 bp before and after the 5’ and 3’ UTRs respectively) as the gene universe. QuickGO Browser [46] (GO version 2023-09-20) was used to generate the ancestor charts for the main GO terms in each group.

## Results

### Differences in global levels of DNA methylation between wild and domesticated grapevine genotypes

**Fig. 1:**
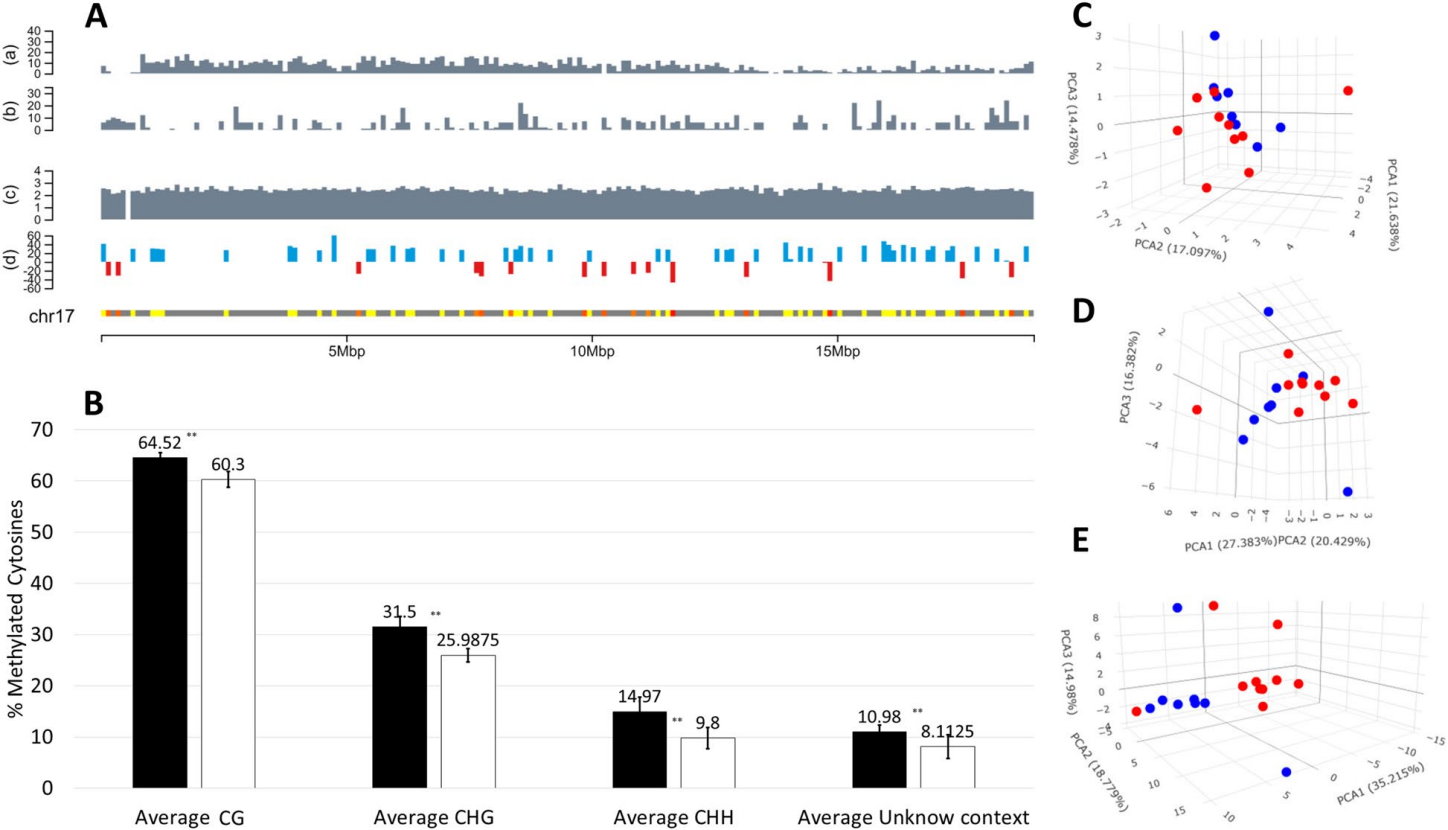
Analysis of differences in global levels of DNA methylation in cultivated andwild *V. vinifera * accessions. **A** Visualization of genomic and epigenomic information for chromosome 17 of *Vitis vinifera* using 100,000 bp windows. Vertical bars in panels (a) and (b) show the number of protein coding genes and transposable elements respectively per genomic window. Bars in panel (c) shows average sequencing depth per genomic window (Log 10 of calculated depth for sequenced bases). Panel (d) shows the average fold change in methylation in given window (blue and red bars indicate an average hypermethylated or hypomethylated window in cultivated vs. wild accessions. Panel containing chromosome number (i.e., chr17 here) shows average fold change in methylation in each window (hypomethylation (orange) hypermethylation (yellow). To visualize an interactive version of the figure containing all DMCs per window in all chromosomes see Supplementary File 1 (Follow instructions available in Supplementary File 2). Panels generated using ChromoMap R [41]. **B** Bars show the average percentage of methylation per sequence context (CG, CHG, CHH, and unknown) in cultivated (*V. vinifera* ssp. *vinifera* (*n* = 10); black bars), and wild type (*V. vinifera* ssp. *sylvestris* (*n* = 8); white bars) accessions. Error bars indicate the calculated Standard Deviation. ** T-test, *p*-value < 0.01. **C-E** Multivariate analysis of percentage of methylation for all individual cytosine sequenced in cultivated and wild *V. vinifera* accessions. Principal Component analysis plots show results for methylation analysis results in the CG (C), CHG (D), and CHH (E) contexts. Blue and red circles represent cultivated and wild accessions respectively. PCs 1 to 3 represent 53, 64, 69% of the total measured variability in CG, CHG, and CHH contexts respectively

 EpiGBS2 libraries yielded a total of 44.5 million reads with an average of 2.5 million reads per sample (ranging from 1,106,659 to 8,249,031 reads). Bisulfite conversion efficiency showed on average 90% unmethylated cytosines converted to uracils. The mean percentage of mappable reads per sample after de-multiplexing was 49%, ranging from 37 to 60%. This resulted in an overall genome coverage of 1.5% (ranging between 0.7% and 2.6% (Supplementary Table S1)), with reads distributed evenly across the whole genome (See Fig. 1A for read distribution across chromosome 17 and Supplementary File 1 for read distribution across all chromosomes).

Methylation calling identified a total of 222,647 genomic locations containing methylated cytosines. The CG context presented the highest level of cytosine methylation, followed by CHG and CHH context (Fig. 1B). Cultivated varieties presented consistent significantly higher (T-test, *p*-value < 0.01) levels of DNA methylation than wild accessions in all sequence contexts (Fig. 1B). PCA plots built using the percentage of methylation for all sequenced cytosines as variables, show that wild and cultivated form two different clusters separated mainly by PC1 in all sequence contexts (Fig. 1C-E). Such observed separation between wild and cultivated accessions is particularly evident for the CHH context (Fig. 1E and Supplementary Fig. 3).

Analysis of global methylation levels in wild and cultivated accessions at genomic feature level (i.e., intergenic and genic regions) showed that intergenic regions presented similar levels of DNA methylation to those observed genome-wide in all sequence contexts, with the exemption of CHGs, which showed higher levels of DNA methylation **(**Supplementary Fig. 1**)**. Conversely, genic regions showed consistently lower levels of DNA methylation in all sequence contexts than those observed genome-wide (Supplementary Fig. 2). Finally, cultivated accessions presented significantly higher levels of DNA methylation (T-test, *p*-value < 0.01) than wild accessions in all sequence contexts and genomic features (Supplementary Figs. 1 and 2).

### Identification of differentially methylated cytosines associated to domestication

**Fig. 2:**
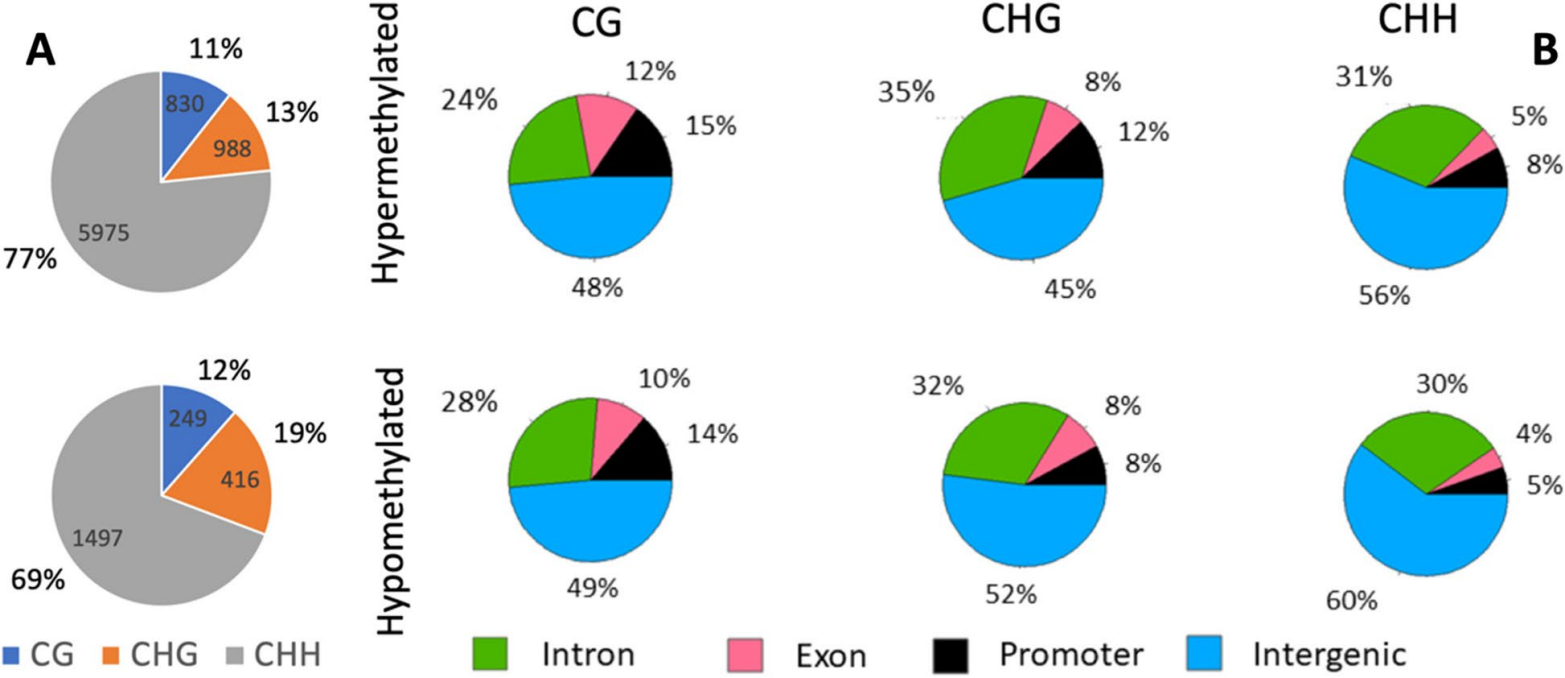
Identification of DMCs associated to grapevine’s domestication. Pie charts show (**A**) the total number and percentage of hypermethylated (top pie chart) and hypomethylated DMCs identified in cultivated vines compared to wild accessions in each sequence context (CG, CHG and CHH); and (**B**) the percentage of DMCs identified per genic feature and sequence context, in cultivated compared to wild type accessions

Differential Methylation analysis identified a total of 9955 DMCs between wild and cultivated accessions evenly distributed across the genome (Fig. 1A and Supplementary File 1). Of those, 7793 DMCs were hypermethylated and 2162 DMCs were hypomethylated in cultivated vines compared to wild accessions. The majority of both hyper and hypomethylated DMCs were found in the CHH context (77 and 69% respectively) (Fig. 2A). From a gene feature context, DMCs were mainly found in intergenic regions **(**Fig. 2B**)**. This is particularly evident in the CHH context, where 56 and 60% of hypermethylated and hypomethylated DMCs, respectively, were found in intergenic regions. The second most abundant genic feature presenting DMCs were introns, with percentages varying between 24 and 35% in hypermethylated DMCs, and 28 and 32% in hypomethylated DMCs, depending on the sequence context **(**Fig. 2B**)**.

### Effect of genetic differences to epigenetic differentiation between wild and cultivated accessions

The EpiDiverse-SNP pipeline identified 57,489 SNPs in the 222,711 genomic locations containing methylated cytosines. Of the remaining 165,189 genomic locations containing methylated cytosines, 5869 DMCs were hypermethylated and 1575 DMCs were hypomethylated in cultivated vines compared to wild accessions (i.e., 25% of the original DMCs were associated to a SNP). Hierarchical clustering analysis using all epialleles and only those not associated to SNPs showed no significant clustering differences (Supplementary Fig. 3).

### Analysis of (epi)genetic signals of provenance in wild type accessions

**Fig. 3:**
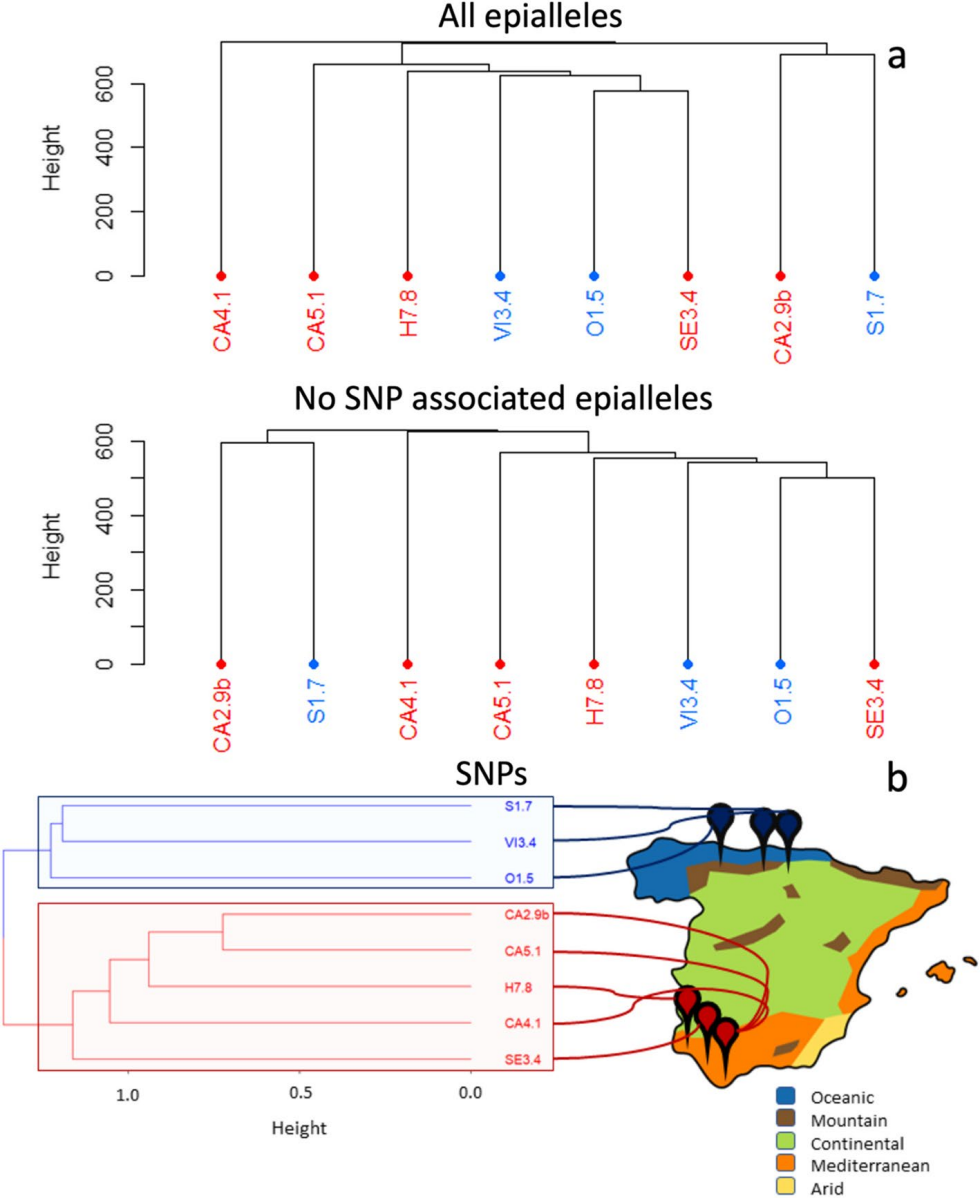
Effect of region of origin on the methylome of Iberian *Vitis vinifera* ssp. *sylvestris.* Analysis of genetic (**a**) and epigenetic (**b**) differences among wild grapevine accessions originally collected from different regions of the Iberian Peninsula and grown in a common garden. Epigenetic analysis was performed using epialleles not associated to SNPs the epiDiverse-SNP pipeline to remove the effect of underlying genetic variation between wild grapevine populations. Samples highlighted in red, the represented branches correspond to wild accessions belonging to the South of Spain, and in blue, they correspond to wild accessions coming from the North of Spain, placed approximately to the map of the Spanish Climate Zones

We then compared wild accessions to determine if a genetic and or epigenetic signal associated to the location from where they were originally collected exist. Hierarchical cluster analysis showed no clear epigenetic signal irrespective of the use of all epialleles sequenced or after removing epialleles associated to a SNP (Fig. 3a). However, when only genetic information was used (i.e., clustering samples using the SNPs identified by the EpiDiverse-SNP pipeline, two separate clusters of wild accessions grouped by their provenance. One cluster contained all three accessions originally collected in the North of the Iberian Peninsula, in oceanic, continental and mountain climatic zones, while the second cluster contained all accession collected from the South of the Iberian Peninsula (Mediterranean climatic zone) (Fig. 3b) (see in Supplementary TableS1 for metadata associated to each accession).

### Analysis of domestication associated DMCs within genic features

**Fig. 4:**
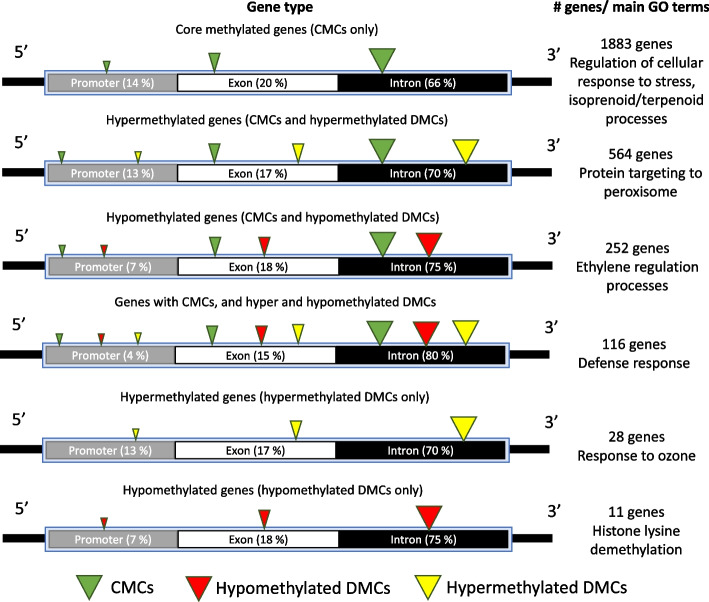
Schematic representation of methylated gene types in wild and cultivated grapevines. Boxes within gene models show the percentage of the total methylated cytosines in each gene group found in each genic context. Arrow heads color and size indicate the type of methylated cytosine found in each gene type (Core methylated cytosines (CMCs); Hypomethylated and hypermethylated cytosines in cultivated vs. wild grapevine accessions) and the abundance of that type of methylation within that gene type, respectively. Right panel shows the number of identified genes for each group and their correspondent most representative GO terms

Collectively epiGBS2 results generated reads overlapping with a total of 7174 genes. Of those, a total of 2854 (40%) genes were identified as genes that contained at least one methylated cytosine (Supplementary Table S2A). Methylated cytosines were mainly found in introns (66–80%), followed by exons (15–20%), and promoters (4–14%) (Fig. 4) (Supplementary Table S2B). Genes containing methylated cytosines could be further divided into six groups, in order of abundance, (1) Genes presenting methylated cytosines both in wild and cultivated accessions (1883 genes) (core methylated genes (CMCs) hereafter); (2) genes presenting CMCs and hypermethylated DMCs in cultivated compared to wild accessions (564 genes); (3) genes presenting CMCs and hypomethylated DMCs in cultivated compared to wild accessions (252 genes); (4) genes presenting CMCs and both hypomethylated and hypermethylated DMCs (116 genes); (5) Genes presenting hypermethylated DMCs in cultivated compared to wild accessions (28 genes); and (6) genes presenting hypomethylated DMCs in cultivated compared to wild accessions (11 genes). Functional analysis of the genes identified within each group revealed that CMCs are significantly associated with the regulation of cellular response to stress and isoprenoid/terpenoid processes. Cultivated grapevines hypermethylated genes were associated mainly to processes associated to protein targeting to peroxisomes and histone lysine demethylation, while hypermethylated genes in wild grapevines related to ethylene regulation processes and response to ozone. The remaining group (i.e., genes both presenting hyper and hypomethylated cytosines between in both types of accessions) presented GO terms related to defense response (Fig. 4) (See Supplementary Table S2C for a complete list of GO terms in each group).

## Discussion

While significant strides have been made in understanding the genetic underpinnings of crop domestication, there is still a relative paucity of knowledge regarding the role of epigenetic mechanisms in this process. Epigenetics has emerged as a crucial regulator of various biological processes in both plants and animals. Recent studies have begun to hint at the potential involvement of epigenetic changes in the adaptation and phenotypic diversification of domesticated crops. However, a comprehensive understanding of how these epigenetic modifications may have been harnessed—or inadvertently altered—during the domestication process is still in its infancy. Studying the contribution of epigenetic mechanisms to domestication [47–49] will provide novel insights into the early stages of domestication and the selective pressures faced by ancestral agriculturists. At the same time, such studies will lay the foundation for the development of comprehensive models integrating plant adaptation to the environment through epigenetics mechanisms, facilitating their use for the development of novel cultivars more resilient to stress [33].

### Epigenetic signal of domestication is independent of genetic variation

Previous studies have shown that DNA methylation variability in plants can be attributed to three main factors: genetic (sequence) differences, environmental induction, and stochasticity (see Konate et al., 2020 for a recent example) [50]. Moreover, Xie et al., showed that cultivation method also contributes to environmentally induced epigenetic variability in grapevines [21]. Our results show significant epigenetic differences between cultivated and wild grapevine accessions, both at a global and genomic feature level. These differences were maintained even after removing all epialleles associated with genetic variability (considered the most abundant type of epigenetic variability [51]). Interestingly, the number of DMCs associated to genetic variation in this study (25%), was very similar to the proportion of DMRs associated to soybean domestication that could be explained by local genetic variation (22.4%) [31]. Since all plants included in this study were grown under the same conditions, this suggests that the remaining epialleles could be considered true epimutations (i.e., not the result of the interaction of the epigenotype with the genotype or with the environment [51]). Such differences were particularly abundant in the CHH and CG contexts. This indicates that different factors might be influencing the methylation of cytosines in different sequence contexts, suggesting context specific selection pressures imposed by domestication. Conversely, DMCs between wild and cultivated accessions were found evenly distributed across all chromosomes, suggesting that no specific epigenomic region has been under special selection. This could be due to the combination of two factors, the reduced representation methylome sequencing of the approach used in this study, and that DNA methylation might be under weak selection during domestication as previously observed in maize [32]. A more detailed analysis using whole methylome sequencing is required to validate this hypothesis in grapevine.

Multiples studies have indicated the relationship between geographic origin and genetic differences in grapevine [7, 43, 52, 53], which would support the premise that genetic induced epigenetic differences should be observable between grapevines genotypes, independently of how long those accessions have been removed from their original source. However, it is not clear, if environmentally induced epigenetic variability is stable over time. To shed light over that question, we compared the methylome of wild grapevine accessions originally collected in different regions of the Iberian Peninsula, which have been maintained under the same growing conditions over 18 years. Although a clear genetic signal of provenance was observed in the wild accessions included here, no epigenetic differences were found between plants originally collected from populations in the North and South of the Iberian Peninsula.

### Grapevine domesticated accessions present higher global levels of DNA methylation

Cultivated accessions consistently exhibit significantly higher global levels of DNA methylation across all sequence contexts and all genomic features. These observations, are in contradiction with previous studies showing that domestication induces a significant decrease in DNA methylation in rice [29] and tomato [30]. This is perhaps explained by the differences in the type of propagation used during the domestication of each species (i.e., vegetative vs. sexual propagation), which could have resulted in diametrically opposed domestication epigenetic syndromes [2, 6]. In fact, the historical use of vegetative propagation in cultivated grapevines [5, 8], has been shown to preserve environmentally induced epigenetic variability in vegetatively propagated perennials [54]. Having noted this, it’s crucial to consider that the observed differences in DNA methylation may not solely result from domestication. Instead, they could stem from the distinct reproductive strategies (hermaphroditic in cultivated accessions versus dioecious in wild accessions) of the plants under study. Irrespective of the driver of the hypermethylation observed in cultivated/clonally propagated accessions, it is also tempting to speculate that the selection for hypermethylation during domestication, might have contributed to the appearance of genetic mutations leading to novel phenotypes, since mutation ratio is higher in methylated cytosines, as previously proposed for clone diversity in grapevines [55].

As seen before [31], in our study a large proportion of DMCs between wild and cultivated accessions were found within intergenic regions. Previous work has suggested that intergenic epialleles might be related to the regulation of long intergenic non-coding RNAs (lincRNAs), which are highly prevalent in the intergenic regions of plant genomes and are found to regulate essential biological processes [56]. The role of long non-coding RNAs (lncRNAs) in crop domestication has become increasingly evident through recent genomic studies. Comprehensive genome-wide analyses identified conserved lncRNAs closely associated with traits selectively enhanced during the domestication process of rice (i.e., panicle architecture, seed-setting rate, grain weight, grain size, and grain composition), highlighting their potential role in trait selection and crop improvement [57, 58]. This body of research suggests that lncRNAs could be significant in modulating gene expression and phenotypic traits crucial for the adaptation of domesticated crops to human agricultural needs. Additionally, a multispecies review on the emerging roles of lncRNAs in agriculture stresses their importance in regulating seed traits that are vitally important for crop yield and quality, with a specific focus on how these molecules contribute to the evolution and refinement of such traits during domestication, and how lncRNAs have the potential to be used for the improvement of agriculturally important seed traits [59]. Collectively, these studies underscore the profound impact of lncRNAs on the genetic architecture of domesticated crops, potentially offering new avenues for enhancing crop performance through biotechnological interventions. Apart from the non-coding RNA elements, it is also possible that the accumulation of methylation in intergenic regions could be related to silencing repeat elements or somatic mutations, which are a major driver of cultivated grapevine genome diversification [54].

Genic regions consistently presented lower levels of DNA methylation in all sequence contexts than intergenic regions, which is a common feature in plant methylomes (see [60] for an example). Nonetheless, 40% of the genes sequenced here presented methylated cytosines. Of these, 1883 (67%) presented only CMCs, i.e., cytosines which were consistently methylated both in wild and cultivated accessions, while the remaining 33% presented CMCs and or DMCs. Of these, a large majority (73%) presented some form of hypermethylation in cultivated compared to wild accessions.

CMCs and DMCs identified within genes were preferentially found within introns, followed by promoters and exons, irrespective of the sequence context. This positional distribution of methylated cytosines around and within genes revealed different strategies in the methylation of genic features associated to the domestication process. In the context of plant promoters, methylation usually acts to repress gene transcription, thereby controlling the timing and spatial patterns of gene expression throughout development and in response to environmental stimuli [61]. In introns and exons, DNA methylation plays multifaceted roles. In exons, DNA methylation is associated with increased gene expression in certain contexts, although the exact mechanism is not fully understood [62]. Within introns, DNA methylation has been shown to influence alternative splicing, whereby different mRNA isoforms are generated from a single gene [62]. In grapevines, alternative splicing has been linked to phenotypic specificities and distinct adaptive capacities by enabling a diverse range of proteins to be produced [63]. Several studies suggest that crop domestication has selected alternative splicing variation linked to desired traits, such as flower production [64], anthocyanin accumulation [65] or cell wall degradation in fruits [66]. Research highlighted in a study on pear (*Pyrus pyrifolia*) revealed how domestication has led to changes in the alternative splicing of genes that contribute to the fruit traits such as sugar metabolism, acid metabolism, stone cell formation, and fruit firmness [66]. Similarly, comparative analyses between wild and cultivated tomato species have shown that domestication impacts environment-responsive alternative splicing in the inflorescences, suggesting a role in adaptability and phenotypic diversity [64]. In the spiny Solanum group, a study identified a DFR gene where alternative splicing, influenced by a natural promoter variant, plays a crucial role in anthocyanin accumulation, a trait selectively enhanced during domestication [65]. Additionally, research on wheat has documented the complex interplay between domestication, polyploidization, and alternative splicing, indicating significant modifications in splicing patterns that may contribute to phenotypic changes and stress responses [67]. Together, these studies underscore the profound influence of domestication and DNA methylation on alternative splicing, driving the evolution of desirable traits in crop species. This intricate interplay between methylation and the genic landscape establishes a regulatory network that finely tunes gene expression and maintains genomic stability, underpinning the complexity and adaptability of plant life.

### Gene specific differential methylation associated to domestication is enriched in response to stress

Functional analysis of methylated genes showed that genes related to important agronomic traits exhibited significant DNA methylation level variation during grapevine domestication, particularly terms associated with stress response. Genes with differential methylation in the form of hypermethylation or hypomethylation between wild and cultivated grapevines were less abundant but still significant. The hypomethylated genes in cultivated grapevines were tied to protein targeting to peroxisomes and histone lysine demethylation. These processes are essential for cellular homeostasis and epigenetic regulation, suggesting that the domestication process may have enhanced or refined these functions in cultivated varieties. Interestingly, Histone H3-K4 demethylation, and DNA hypermethylation, have both been associated with gene expression repression [68]. Moreover, genes hypermethylated in wild grapevines were found to relate to ethylene regulation processes and response to ozone. Ethylene is a critical hormone in plants, mediating various stress responses [69]. In grapevine, ethylene signaling plays a crucial role beyond managing abiotic stress, encompassing various agronomically important traits such as bud dormancy and berry development. For bud dormancy, research has shown that a transient induction of specific ethylene biosynthesis genes may be involved in the regulation of the release of grapevine bud dormancy, indicating a targeted genetic response that mediates dormancy transitions [70]. Further studies identified potential events following ethylene signaling that are triggered by stimuli promoting bud dormancy release, suggesting a complex network of regulatory mechanisms [71]. In the context of berry development, interactions between ethylene and auxin have been identified as pivotal in controlling the ripening process of grape berries. This interaction points to a synergistic action between these hormones that is crucial for fine-tuning the developmental processes that lead to optimal fruit maturation [72]. These insights collectively enhance our understanding of ethylene’s multifaceted role in grapevine biology, influencing both growth cessation and fruit development. Moreover, recent studies have highlighted the interplay between DNA methylation and ethylene-responsive genes under stress conditions and their relationship with ABA in regulating bud dormancy. For instance, in woodland strawberry, dynamic changes in DNA methylation have been observed in response to stress, affecting genes including those responsive to ethylene, which are crucial for adaptation and survival [73]. Moreover, research on perennials has demonstrated that ABA plays a significant role in bud dormancy, where changes in DNA methylation patterns might regulate the expression of ethylene-responsive genes critical for dormancy initiation and release [74]. These findings suggest that methylation changes in ethylene-responsive genes are a key mechanism through which plants modulate developmental and stress-related responses, presenting a fertile area for future research into crop improvement and adaptation strategies.

The unique category of genes that showed both hyper and hypomethylated cytosines in both types of accessions, albeit being the smallest group, associated with defense response. Intriguingly, core methylated genes (CMCs) were also associated with stress response. This multimodal pattern of methylation during grapevine domestication suggests a complex regulation mechanism and might hint at genes that have retained some functionality from their wild origins, while also adapting new functionalities for the domesticated environment. Also, the conservation of methylation in the core methylated genes could suggest that the functions they support are essential and have remained unchanged between wild and cultivated grapevines.

## Conclusions

In summary, our research provides compelling evidence that there are significant differences in DNA methylation patterns between wild and cultivated grapevines. These differential methylation patterns between the two types of grapevine accessions offer intriguing insights into the potential origin and roles of DNA methylation in their divergence. The observed prevalence of hypermethylated DMCs in cultivated grapevines across all contexts (CG, CHG, and CHH), underscores our hypothesis that cultivated grapevines accrue more DNA methylation than their wild counterparts. The varied associations of these methylation patterns to vital processes such as alternative splicing, stress response, hormone regulation, and defense mechanisms underscore the potential implications of epimutations in shaping the evolutionary and developmental trajectories of domesticated species, influencing in the crop’s plasticity and uniformity. Nevertheless, since this study only included hermaphrodite flower producing cultivated accessions, further studies including dioecious cultivated accessions are required to determine if the epigenetic differences identified here are really associated with domestication or to the sexual strategy of the studied plants. Additionally, future studies should analyze complete methylomes and focus on the consequences of methylation changes on gene expression to gain a comprehensive understanding of the role of DNA methylation in grapevine domestication.

### Supplementary Information

Supplementary Material 1.

Supplementary Material 2.

Supplementary Material 3.

Supplementary Material 4.

Supplementary Material 5.

Supplementary Material 6.

Supplementary Material 7.

## Data Availability

The datasets generated and analyzed during the current study are available in the European Nucleotide Archive (ENA), accession number PRJEB55284.
