# Supplementary material for: Epigenetic differences between wild and cultivated grapevines highlight the contribution of DNA methylation during crop domestication
Source: BMC Plant Biol. 2024 Jun 6;24:504. doi: 10.1186/s12870-024-05197-z (PMC11155169; doi:10.1186/s12870-024-05197-z)
Supplement: Supplementary file 2 — Supplementary Material 2. [file 12870_2024_5197_MOESM2_ESM.docx]

**Supplementary File 1**: **Interactive visualization of genomic and epigenomic information for all chromosomes of *Vitis vinifera* using 100,000 base pair windows.** Vertical bars in panels (a) and (b) show the number of protein coding genes and transposable elements respectively per genomic window. Bars in panel (c) shows average sequencing depth per genomic window (Log 10 of calculated depth for sequenced bases). Panel (d) shows the average fold change in methylation in given window (blue and red bars indicate an average hypermethylated or hypomethylated window in cultivated vs wild accessions. Panels containing chromosome number show average fold change in methylation in each window (hypomethylation (orange) hypermethylation (yellow). To visualize all DMCs per window hover the cursor over the desired window. A pop-up display with detailed information (i.e., Chromosome coordinates of the window within the chromosome (in base pairs), methylation status (hypomethylation (orange) hypermethylation (yellow) and IDs of DMCs within the window, number of DMCs (counts) within the window, average, maximum, and minimum fold change in methylation of all DMCs with in the window). Panels generated using ChromoMap R (41).
