# Supplementary material for: Epigenetic differences between wild and cultivated grapevines highlight the contribution of DNA methylation during crop domestication
Source: BMC Plant Biol. 2024 Jun 6;24:504. doi: 10.1186/s12870-024-05197-z (PMC11155169; doi:10.1186/s12870-024-05197-z)
Supplement: Supplementary file 5 — Supplementary Material 5. [file 12870_2024_5197_MOESM5_ESM.pdf]

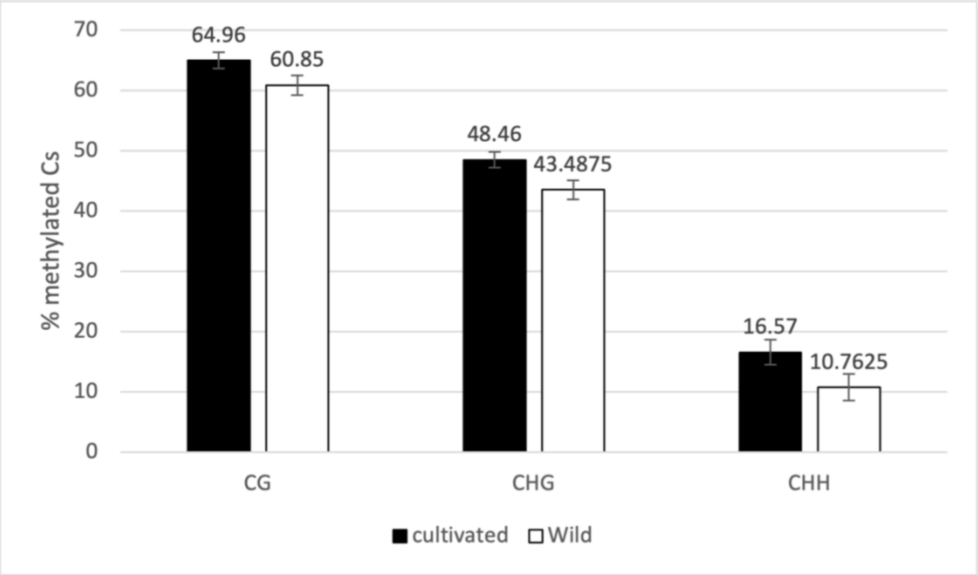

Supplementary Figure 1: **Analysis of differences in global levels of DNA methylation in intergenic regions of cultivated and wild *V. vinifera* accessions.** Bars show the average percentage of methylation per sequence context (CG, CHG, and CHH) in cultivated (*V. vinifera* ssp. *vinifera* (n = 10); black bars), and wild type (*V. vinifera* ssp. *sylvestris* (n = 8); white bars) accessions. Error bars indicate the calculated Standard Deviation. ** T-test, p-value < 0.01.
