# Supplementary material for: Epigenetic differences between wild and cultivated grapevines highlight the contribution of DNA methylation during crop domestication
Source: BMC Plant Biol. 2024 Jun 6;24:504. doi: 10.1186/s12870-024-05197-z (PMC11155169; doi:10.1186/s12870-024-05197-z)
Supplement: Supplementary file 7 — Supplementary Material 7. [file 12870_2024_5197_MOESM7_ESM.pdf]

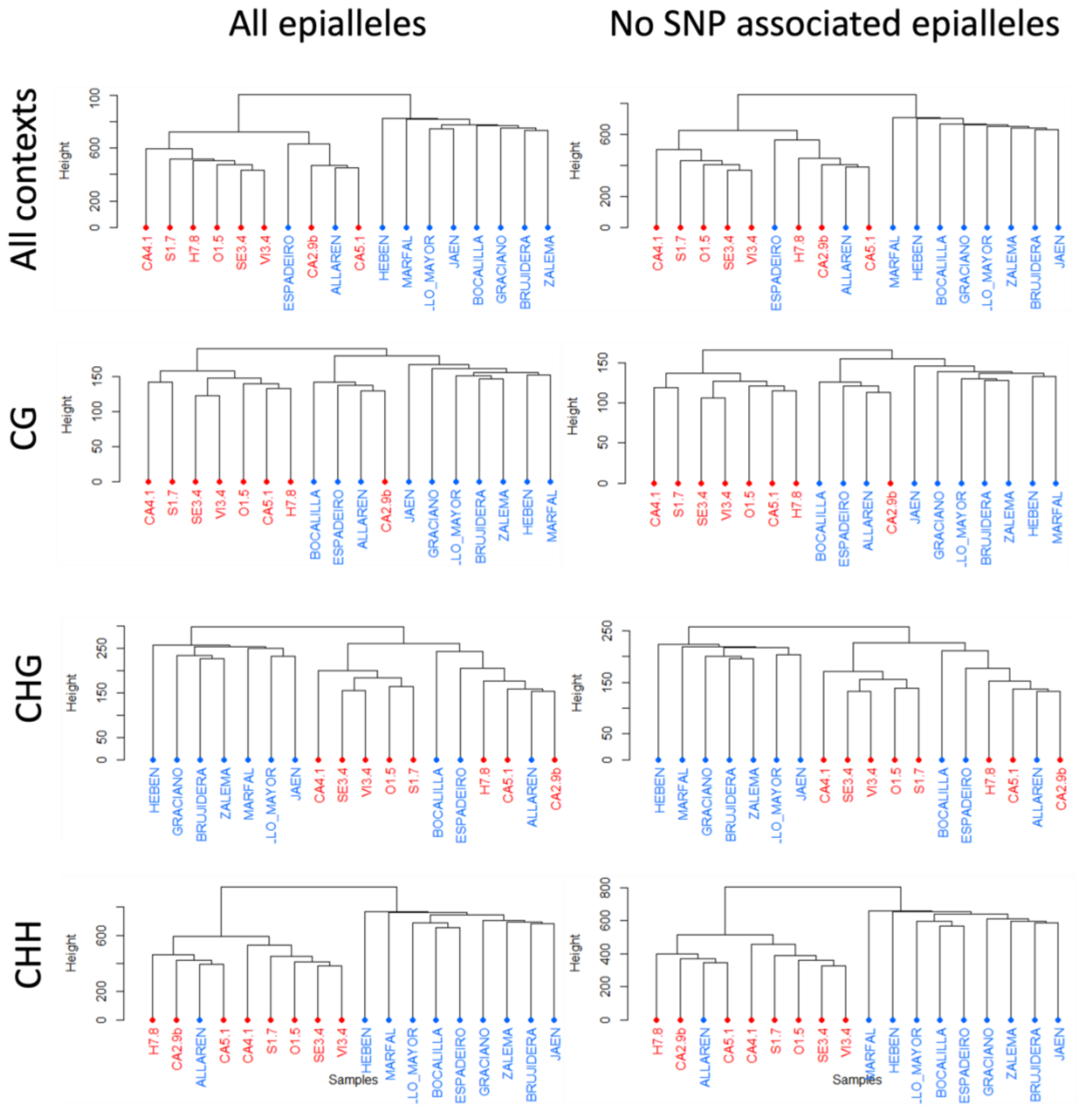

Supplementary Figure 3. Analysis of differences in global levels of DNA methylation in cultivated and wild V. vinifera accessions. *Hierarchical cluster analysis of percentage of methylation for all 222,711 epialleles sequenced (left column), or 165,189 epialleles not associated to SNP (right column), in cultivated (blue) and wild (red) V. vinifera accessions. Cluster plots show results for methylation analysis results containing all sequence context, and separated by context (e.g., CG, CHG, and CHH).*
